# Pre-dental treatment screening in Indonesia during the COVID-19 pandemic: a questionnaire survey of dental practitioners

**DOI:** 10.1186/s12903-023-03004-z

**Published:** 2023-05-22

**Authors:** Armelia Sari Widyarman, Moehamad Orliando Roeslan, Iwan Dewanto

**Affiliations:** 1 Department of Microbiology, Faculty of Dentistry, Universitas Trisakti, Kyai Tapa 260, Grogol, 11440 West Jakarta Indonesia; 2 Department of Oral Biology, Faculty of Dentistry, Universitas Trisakti , Kyai Tapa 260, Grogol, 11440 West Jakarta Indonesia; 3 Faculty of Medical and Health Science, School of Dentistry, University Muhammadiyah Yogyakarta, Bantul, 55183 Indonesia

**Keywords:** COVID-19, Pre-procedure, Dental treatment, Indonesia, Dentist, SARS-CoV-2

## Abstract

**Background:**

Dental practitioners have a high risk of contracting COVID-19 during the treatment of patients because of exposure to airborne droplets. However, the application of pre-procedure treatment screening in dental practices in Indonesia varied during the pandemic. The purpose of this study was to investigate the use of updated pre-procedure dental treatment protocols and procedures among dental practitioners in Indonesia.

**Methods:**

This study consisted of dentists registered as members of the Indonesian Dental Association who attended the Indonesian Dental Association webinar series in 2021. All the participants completed a questionnaire survey. The participants, who were from various regions in Indonesia, were granted password-protected access to a URL hosting the questionnaire. The questionnaire collected demographic information and contained questions on adherence to updated protocols and patient screening procedures, to which the respondents answered “Yes” or “No”. For the analysis, the participants were divided into three groups based on the type of facility where they were employed: public (government) hospitals, private hospitals, or university hospitals (dental schools). A chi-square test was used to investigate the association between professional background and the implementation of updated protocols, including pre-procedure dental treatment screening. A value of *P* < 0.05 was considered statistically significant.

**Results:**

The age range of the participants was 20 − 60 years. The participants worked in facilities in 32 provinces in Indonesia. In total, there were 5,323 participants (males: *n* = 829; females: *n* = 4,494). In terms of professional backgrounds, 2,171, 2,867, and 285 participants were employed in government hospitals, private hospitals, and dental faculties, respectively. Among 5,232 participants who implemented updated COVID-19 prevention protocols, 5,053 (98%) participants performed pre-surgery procedures Among 151 participants who did not implement updated COVID-19 prevention protocols, 133 (88%) individuals carried out pre-rinse procedures.

**Conclusions:**

Almost all the dental practitioners employed in government hospitals, private hospitals, and dental faculties in Indonesia performed pre-surgery patient screening procedures. There was an agreement between the dental professionals in all three settings on the need for COVID-19 pre-treatment screening procedures in dental practices during the COVID-19 pandemic.

## Introduction

Coronavirus disease 2019 (COVID-19) is a novel disease. World Health Organization (WHO) had labeled COVID-19 as a global pandemic [1]. As of 17 September 2021, there were 226,884,344 confirmed cases worldwide, including 4,666,334 deaths [2], making the pandemic one of the deadliest in history [3]. On this date in Indonesia, there were 4,185,144 confirmed cases, including 140,138 deaths [2]. Since the pandemic, many preventive measures aimed at to maintain and containing the COVID-19 pandemic. These include social distancing, wearing face masks in public spaces, air filtering, surface disinfection, and quarantining symptomatic people [4].

Coronavirus disease 2019 (COVID-19) is caused by a virus called severe acute respiratory syndrome coronavirus 2 (SARS-CoV2) that has been linked to the previous SARS coronaviruses and bat coronaviruses [5]. SARS-CoV2 spread mainly via direct methods, such as droplets and aerosols produced by individuals, or indirect methods, such as contact with contaminated objects and airborne contagion. The use of personal protective equipment can serve as an additional source of indirect airborne infection [6]. In general, droplets can travel no more than 2 m and remain infectious only for a short period. However, SARS-CoV2 can remain intact and contagious in the form of droplets in the air for up to 3 h. This unique characteristic of SARS-CoV2 makes it more infectious than its predecessors [7].

According to the Centers for Disease Control and Prevention (CDC), high volumes of aerosols generated in dental practices during patient treatment are a potential source of viral infection. For this reason, dental practices are highly vulnerable to COVID-19 infection [8]. At the beginning of the pandemic, the Indonesia Dental Association published guidelines on disease transmission in dental practices to prevent infection and break the chain of transmission [9]. COVID-19 forced dentists to review their procedures before treating patients to avoid SARS-CoV-2 infection and COVID-19 disease. The mode of transmission of SARS-CoV-2 through droplets and aerosols means that dental practices must implement additional precautionary measures, as the standard of protection that is usually used by dentists is not sufficient to protect themselves and others against SARS-CoV-2 infection [10]. Dentists are expected to update their knowledge of protective equipment to help prevent SARS-CoV-2 transmission [11].

The WHO and CDC recommend that dental clinics perform pre-appointment screening and triaging, including taking temperature measurements and questioning patients about their travel histories [8, 12]. Triaging dental patients using a teledentistry platform, before attending the dental clinic is very useful to verify the COVID-19 risk status of the patient, assess the urgency of the dental condition, and provide self‐care advice when appropriate. Upon telephone triage, if there is no urgency and dental treatment can be delayed, patients and parents should be advised of healthcare instructions and appropriate medication, if required [13]. Subsequently, a body temperature measurement before an individual enters a hospital or dental clinic is one way to detect potential infection. Since a high temperature is a symptom of COVID-19 [14]. If the temperature exceeds 37.3 °C, dental treatment should be postponed [14]. Likewise, information on a patient’s travel history is important, as it enables the dentist to determine whether the patient has traveled to areas with high numbers of COVID-19 cases.

All dentists are obliged to enhance their knowledge of disease prevention, including pre-procedure screening methods, during the pandemic. This includes screening patients before treatment and knowing how to disinfect rooms and dental chairs post-treatment. In addition. dentists must know the proper use and disposal of personal protective equipment, as well as the use of N95 masks, gloves, also donning and doffing [15]. The N95 mask is a certified device to reduce aerosol exposure. It could block 95% of articles of 300 nm [16]. Another strategy to minimize airborne contamination is the use of antimicrobial solutions, and dentists should instruct their patients to rinse their mouths with an antimicrobial mouthwash before all oral procedures [17].

There are 41,003 active dentists throughout Indonesia, some of whom practice in rural areas. To the Indonesian Dental Association (Persatuan Dokter Gigi Indonesia / PDGI) data, there are more than 40,000 dentists, and > 70% of them are women. [18]. Dentists practicing not only in urban areas but also in rural areas need to implement updated COVID-19 prevention protocols and procedures to protect both themselves and their patients. To the knowledge of the authors, no studies have investigated pre-procedure dental treatment screening practices aimed at preventing COVID-19 transmission among dentists in Indonesia. We believe that the implication of such a study is important to shed light on the level of dental practitioners’ knowledge about pre-procedure dental treatment screening, which is very important to control the transmission and prevent a further spike in COVID-19 cases. Thus, the purpose of this study was to investigate the use of updated pre-procedure dental treatment protocols and procedures among dental practitioners in Indonesia.

## Materials and methods

This is a cross-sectional observational study of dental practitioners registered as members of the Indonesian Dental Association as the respondents of this study. Participants who attended four webinar series hosted by the Indonesian Dental Association on the 18th of June, 25th of June, 2nd of July, and 15th of July 2021 completed a questionnaire survey. The Google docs platform was used to create the questionnaire, which was then distributed online. This study describes the perceptions and practice-based awareness of Indonesian dental practitioners during the COVID-19 pandemic. The questionnaire collected demographic information and contained 11 questions divided into two focal areas, namely regarding the dentist’s knowledge based on the dentist’s perception of the COVID-19 transmission procedure and how dental practitioners implemented prevention of COVID-19 transmission in their services. The Questions regarding the implementation of preventing the transmission of COVID-19 by dentists focused on pre-dental treatment (before entering the dentist’s practice) and during dental treatment. All questions are close-end questions, with only two possible answers (“Yes” or “No”). The questionnaire was adapted from Widyarman et al. 2020 and modified to achieve the necessary answers [19]. The modified questionnaire was evaluated for its validity and reliability, with a statistically scaled using Cronbach alpha equals 0.72. Before the actual study, the questionnaire was trialed in 32 educational dental hospitals across Indonesia.

The participants, who were from various regions throughout Indonesia, were granted password-protected access to the URL hosting the questionnaire. A unique study ID ensured the confidentiality of all self-reported data. The participants’ responses were stored in a cloud database, where the data were automatically sorted, scaled, and scored using custom Microsoft Office Excel formulas. This study was approved by the Faculty of Dentistry Trisakti University Ethic Commission no: 011/S3/KEPK/FKG/8/2021. All methods were performed following the relevant guidelines and regulations (Declaration of Helsinki). Following the data collection, the participants were divided into three groups based on where they were employed: government hospitals, private hospitals, and dental faculties.

### Statistical analysis

A chi-square test was used to analyze the associations between the participants’ professional backgrounds and the implementation of updated protocols and pre-procedure treatment screening. IBM SPSS Statistics, version 25 (IBM, Armonk, NY, USA) was used for statistical analysis. A *P* value of < 0.05 was considered statistically significant.

## Results

The age range of the participants was 20 − 60 years. In total, there were 5,323 participants (males: *n* = 829; females: *n* = 4,494) (Table 1). Based on the responses to the questionnaire, 5,232 of the 5,323 had implemented recommended COVID-19 prevention protocols. In the study group, 5,053 (98%) of the participants performed pre-surgery procedures. Of 151 participants who responded that their knowledge of COVID-19 was not up-to-date, 133 (88%) of these respondents were responsible for overseeing *what aspects of patient care*, including pre-rinse procedures. More government hospitals than private hospitals had implemented updated protocols (odds ratio [OR]: 1.6; 95% confidence interval [CI]: 1.1 to 2.2); *P* = 0.005). More government hospitals than private hospitals dental practitioners answered “Yes” to the question: “Do you administer a rapid antigen test (RAT) to all staff in your dental clinic?” (OR: 1.8; 95% CI: 1.6 to 2; *P* < 0.001). In response to the question, “Do all patients undergo a RAT before dental treatment in your dental clinic?” more dental practitioners employed in dental faculties and private hospitals answered “Yes” than practitioners employed in government hospitals (OR: 2; 95% CI: 1.6 to 2.5; *P* < 0.001). In response to the question “Do you regularly perform telemedicine?”, more dentists in government and private hospitals performed telemedicine than dentists in dental faculties (OR: 2.1; 95% CI: 1.6 to 2.7; *P* < 0.001 (Table 2).

**Table 1:** Demographic Table

Category		n	%
Gender	Female	4502	84.58
	Male	821	15.42
Division	Academic	287	5.39
	Private	2866	53.84
	Government	2170	40.77
Region	Riau Islands	109	2.05
	Bangka Belitung Islands	59	1.11
	Aceh	23	0.43
	North Sumatera	80	1.50
	West Sumatera	69	1.30
	South Sumatera	93	1.75
	Bengkulu	9	0.17
	Jambi	33	0.62
	Lampung	44	0.83
	Banten	331	6.22
	Jakarta	1092	20.51
	West Java	952	17.88
	Central Java	503	9.45
	Yogyakarta	316	5.94
	East Java	816	15.33
	Bali	104	1.95
	East Nusa Tenggara	34	0.64
	West Nusa Tenggara	17	0.32
	Central Kalimantan	20	0.38
	North Kalimantan	18	0.34
	South Kalimantan	34	0.64
	West Kalimantan	24	0.45
	East Kalimantan	87	1.63
	South Sulawesi	256	4.81
	Central Sulawesi	18	0.34
	West Sulawesi	14	0.26
	Southeast Sulawesi	14	0.26
	North Sulawesi	43	0.81
	Gorontalo	9	0.17
	Maluku	20	0.38
	North Maluku	25	0.47
	Papua	29	0.54
	West Papua	19	0.36
	Outside Indonesia	4	0.08
	No information	5	0.09

**Table 2:** Odds ratios (ORs) and their 95% confidence intervals (CIs) for dental practitioners in government hospitals, private hospitals, and dental faculties

Survey Items	Dental Clinic	OR (95%CI)*	P value
Update protocol questionnaire?	Private	1	
	Government	1.6(1.1 to 2.2)	0.005
	Academician	0.9(0.4 to 2.1)	0.8
Do you check the patient temperature prior to treatment?	Private	1	
	Government	1.1(0.9 to 1.5)	0.3
	Academician	1(0.5 to 1.9)	0.9
Do you always ask the patient travel history as a history protocol?	Private	1	
	Government	1(0.7 to 1.4)	0.9
	Academician	0.6(0.3 to 1.6)	0.3
Are you delaying dental treatment for patient who show symptoms of Covid-19?	Private	1	
	Government	0.7(0.3 to 1.3)	0.2
	Academician	0.4(0.05 to 2.8)	0.3
Do you think N-95 mask should be used in treating dental patient?	Private	1	
	Government	0.9(0.5 to 1.6)	0.8
	Academician	1.6(0.6 to 4.2)	0.3
Do you administer a rapid antigen test (RAT) to all staff in your dental clinic?	Private	1	
	Government	1.8(1.6 to 2)	< 0.001
	Academician	1.2(0.9 to 1.5)	0.2
Do all patients undergo a RAT prior to dental treatment in your dental clinic?	Government	1	
	Private	1.2(1.1 to 1.4)	< 0.001
	Academician	2(1.6 to 2.5)	< 0.001
Do you regularly perform Telemedicine?	Academician	1	
	Government	2.1(1.6 to 2.7)	< 0.001
	Private	1.6(1.2 to 2.1)	< 0.001

**Fig. 1:**
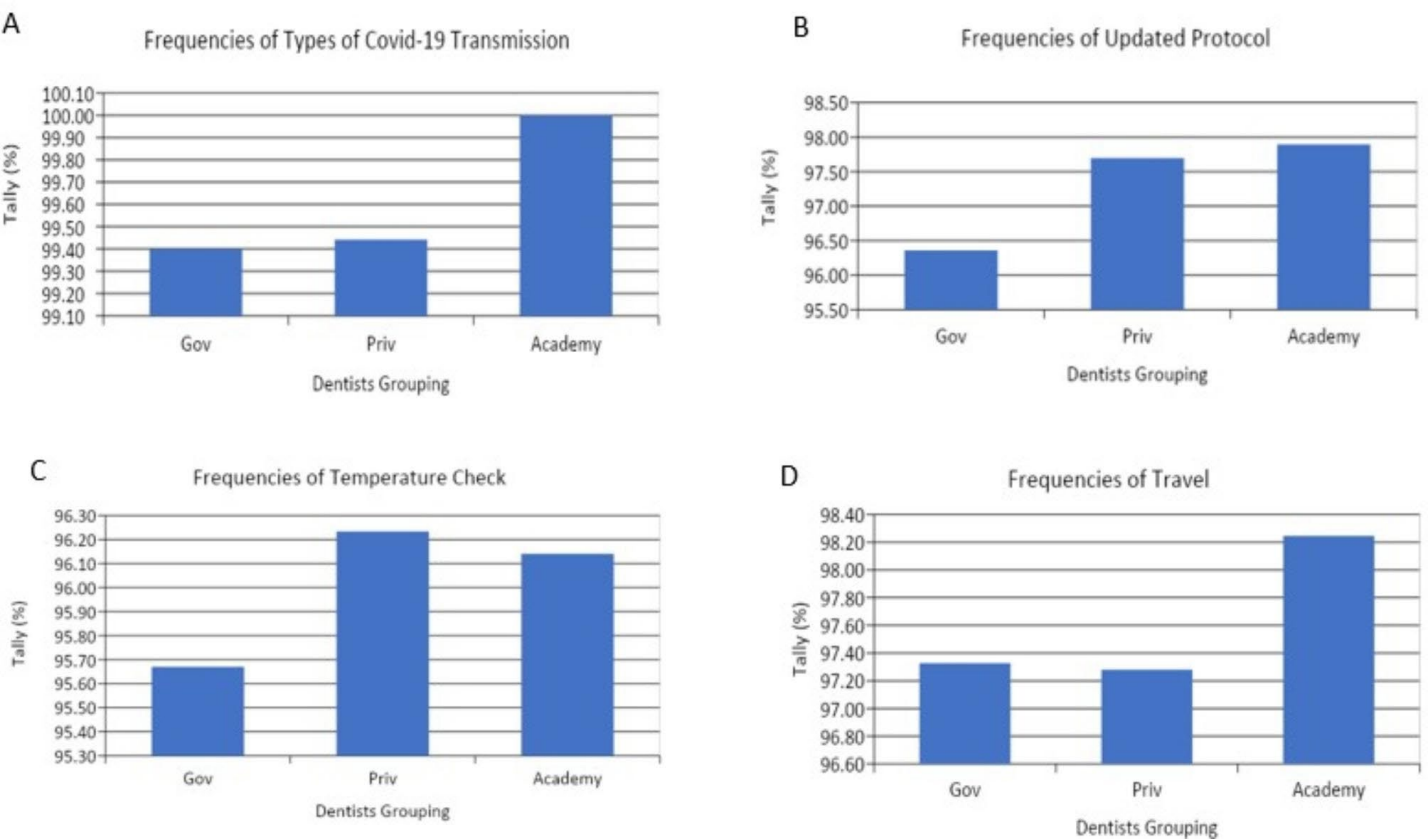
(A) Practitioner’s knowledge regarding the modes of COVID-19 transmission; (B) The number of respondents in different groups who have implemented recommended CDC and WHO protocols in response to the COVID-19 pandemic; (C) Frequency of pre-procedure patient screening (temperature check) by the different groups prior to patient treatment; (D) Frequency of pre-procedure patient screening (travel histories) by the different groups prior to treatment

All dentists who work in university (100%), and almost all dentists who work in government and private hospital (99.4%) knows the modes of Covid-19 transmission (Fig. 1A). Dentists in universities (97.7%), dentists in private hospitals (97.6%), and dentists in government hospitals (96.4%) have updated their pre-procedure protocols before dental treatment according to recommendations by CDC and WHO (Fig. 1B). Dentists in private hospitals (96.2%), dentists in universities (96.1%), and dentists in government hospitals (95.6%) checked their patient’s temperatures before dental treatment (Fig. 1C). Meanwhile, 97–98% of respondents always ask for the travel histories of patients before treatment (Fig. 1D).

**Fig. 2:**
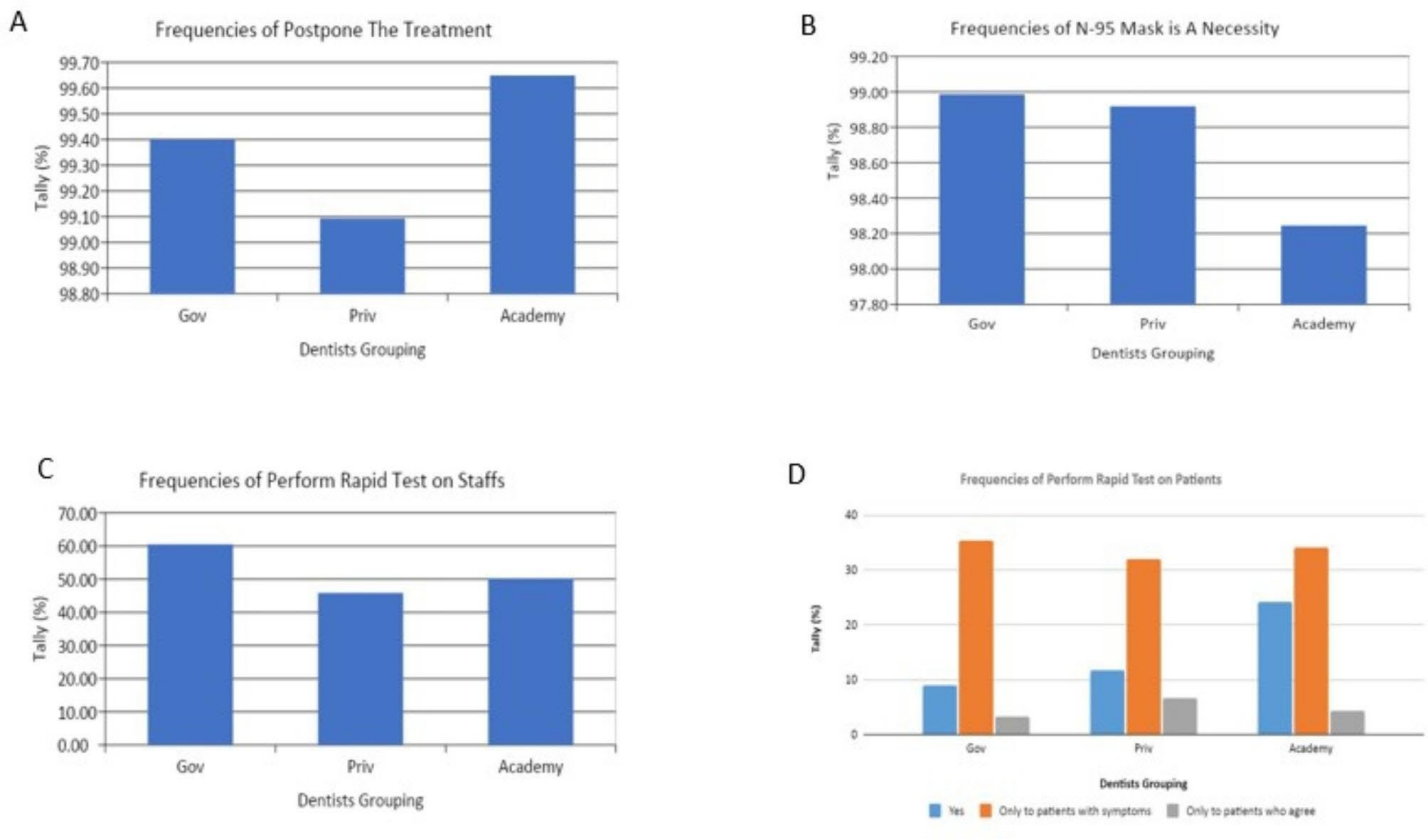
(A) Frequency of postpone dental treatment for patients showing symptoms of COVID-19 by the different groups; (B) Frequency of using N95 mask as a necessity during dental treatment by the different groups; (C) Frequency of rapid Covid-19 testing of staff performed in the different institutions; (D) Frequency of rapid Covid-19 testing of patients performed in the different institutions prior to dental treatment

Most of the dentists from all groups (99%) postponed dental treatment for patients showing Covid-19 symptoms (Fig. 2A). Likewise, with the respondents’ decision to always wear an N95 mask during dental treatment, most of the dentists (98%) from all groups think it is a necessity (Fig. 2B). 60% of dentists in government hospitals performed rapid tests for Covid-19 on staff, while only 50% and 45% of the dentists from university and private hospitals, respectively, performed the rapid test on staff (Fig. 2C). Approximately 30% of respondents from all groups would only conduct rapid tests on patients who have symptoms of COVID-19. 20% of respondents from the university would conduct a rapid test on all patients’ prior dental treatment. Meanwhile, approximately 10% of respondents from government and the private hospital would conduct the rapid test on all patients. Only 10% of respondents would conduct a rapid test on patients who agreed (Fig. 2D).

**Fig. 3:**
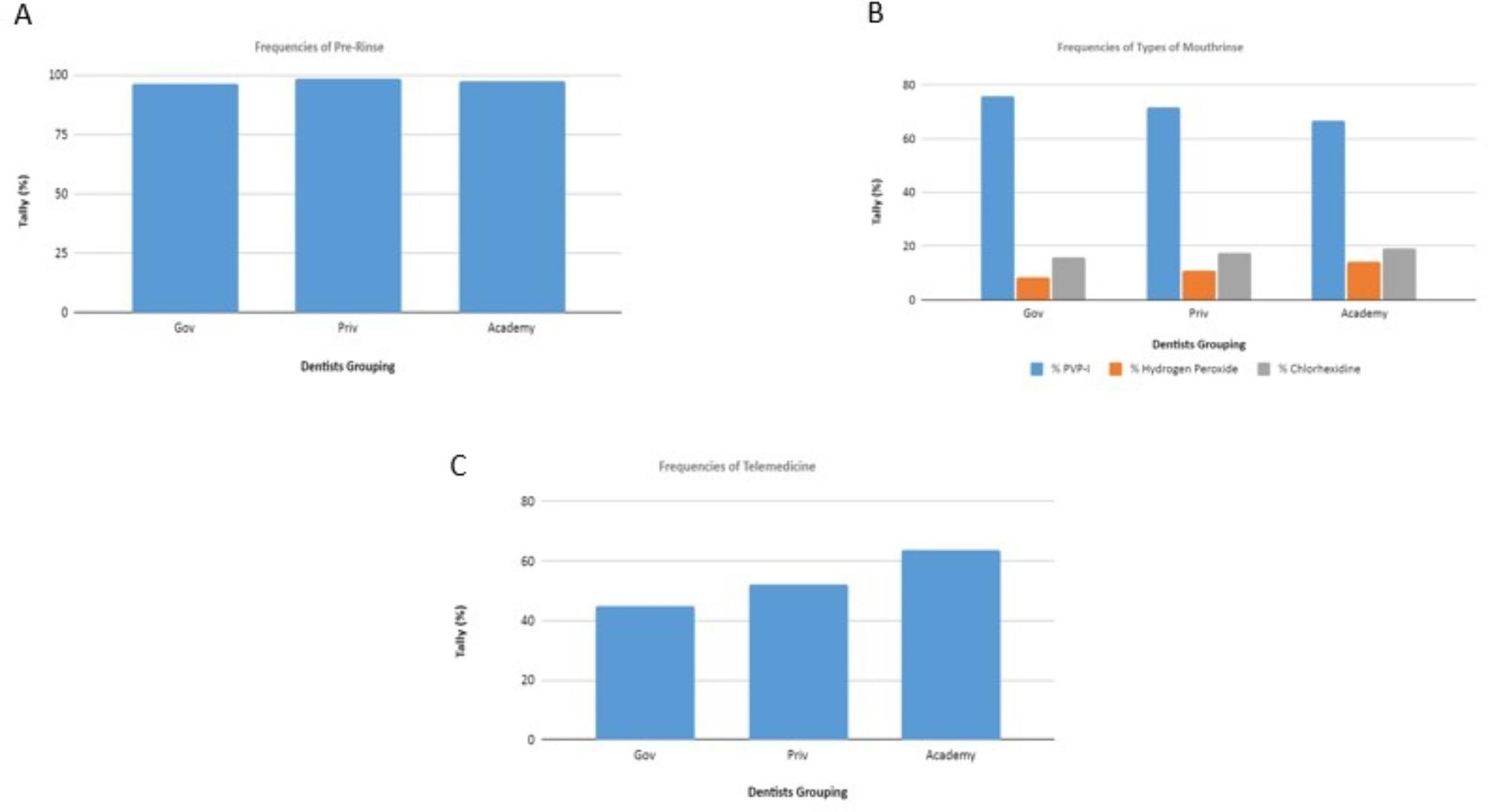
(A) Frequency of pre-rinse with mouthwash prior to dental treatment by the different groups; (B) Frequency of use of different type of mouthwashes by the different groups; (C) Frequency of telemedicine performed by the different groups

Almost all respondents instructed patients to pre-rinse with mouthwash prior to dental treatment (Fig. 3A). Approximately 70% of respondents choose PVP-I as mouthwash, while hydrogen peroxide and chlorhexidine were only chosen by approximately 20% of respondents (Fig. 3B). Approximately 60% of respondents from universities performed telemedicine, while in the other group, only about 50% of respondents performed telemedicine (Fig. 3C).

## Discussion

High volumes of aerosols and droplets are disseminated into the air during dental procedures. As SARS-CoV-2, the virus responsible for COVID-19 is transmitted during human − human interactions, [6] dental health practitioners are highly susceptible to COVID-19 disease. Furthermore, they may serve as carriers of the virus, transmitting the disease to their patient’s dental practitioners need to be aware of the symptoms of asymptomatic COVID-19 disease and implement strict protocols, including pre-procedure patient screening, in dental practices [20]. In this study, we investigated treatment screening procedures and protocols adopted by dentists in three different settings (public, private, and university hospitals) in Indonesia in response to the COVID-19 pandemic.

The results showed that 100% of dentists who work at the university answered the question about the modes of COVID-19 transmission correctly. Most of the dentists (99%) of the dentists employed in government hospitals and private dental clinics were also aware of the various modes of COVID-19 transmission. Almost 98% of dentists who work in university hospitals answered the question about patient care in terms of infection control correctly. This may be because dentists who work in educational institutions feel they must update their knowledge. After all, as teachers, they must transmit up-to-date knowledge to their students. This result is in line with that of a similar previous study [19], which showed that dentists working in educational institutions had better knowledge about the modes of Covid-19 transmission than dentists working in government hospitals and private practices [19].

Infection control plays an important role in preventing the spread of COVID-19. The WHO and CDC have issued recommended protocols for the management of patients so that both dentists and patients are protected during dental treatment [8]. The dentist’s practice room poses a particularly high risk of COVID-19 transmission to dentists, dental assistants, and patients through airborne dissemination of the virus via aerosols or droplets [21]. As noted above, almost all the respondents in the present study were aware of the importance of updating their knowledge about the modes of COVID-19 transmission and infection control. Thus, dentists are aware that their profession poses a high risk of both contracting and transmitting COVID-19.

As shown by the results of this study, almost all the respondents (96%) checked their patients’ body temperatures before dental treatment. Checking a patient’s body temperature before treatment is a useful screening method, as an elevated temperature is one of the symptoms of COVID-19. The body temperature check is usually carried out before entering the building by a building screening officer, but it is common for patients to have their body temperature checked again before dental treatment by a dental assistant as part of pre-procedure screening. The American Dental Association recommends checking a patient’s body temperature as part of an in-office registration procedure using a no-touch forehead temperature scanning device [22].

Currently, there are four regional risk categories related to the spread of COVID-19 in Indonesia: high-risk areas (red zone), moderate-risk areas (orange zone), low-risk areas (yellow zone), and unaffected areas (green zone). Health protocols must be implemented and adhered to in each of these color zones. Information on these protocols can be accessed through the government’s website or a mobile application, and the information is updated daily [23]. By questioning patients about their travel histories before dental treatment, dentists can be aware of potential risks and take steps, including preventive measures, to address these risks. The American Dental Association has recommended always asking patients about their recent travel histories prior to dental treatment as part of pre-procedure dental treatment screening [24].

Almost all the respondents (99%) in the three dental practitioner settings said that they would postpone dental treatment if a patient showed symptoms of COVID-19. This shows that dentists are aware of the importance of precautionary measures to prevent COVID-19 transmission. An increase in the number of dentists who have contracted COVID-19, together with an increase in the number of people infected with COVID-19, means that dentists are more diligent than ever in terms of dental care screening [25]. Thus, dentists have put in place stricter patient screening procedures.

N95 masks are designed to provide protection against airborne particles and aerosols [26]. Their protective ability is due to the presence of four layers of polypropylene and a particle filtration capacity of 0.3 μm. The outer layer is composed of hydrophobic non-woven polypropylene that is moisture resistant. The second and third layers are made of melt-blown nonwoven polypropylene, which captures particles of various sizes through inertial impaction, interception, diffusion, and electrostatic attraction [27]. The innermost layer is composed of moisture-resistant nonwoven polypropylene material [28]. Due to the tight fit of the mask on the user’s face, leakage is improbable [29–31]. Therefore, this mask is highly recommended for dentists during dental treatment as a prevention and infection control method [8]. Almost all the respondents (99%) said they wear an N95 mask during dental treatment. On the other hand, in a similar study on Turkish dentists, of 1,095 respondents, only 38.4% used N95 masks during dental treatment [32]. This may be because at the time the data in the Turkish study were collected, the N95 mask was very new to the market [32]. Furthermore, even if available, the mask was expensive. However, dentists in Indonesia showed different behavior compared to Turkish dentists, who may not even provide dental practice during the pandemic if they do not use an N95 mask.

The results of this study indicate that approximately only 50 − 60% of dentists provide RATs to their staff (Fig. 2C) and 30% of dentists administer RATs to patients with symptoms (Fig. 2D). The RAT, despite its lower accuracy and other limitations compared to quantitative reverse transcription Polymerase Chain Reaction (RT-qPCR) detection, is an efficient and easy-to-use test, which requires no special training for COVID-19 detection. Providing RATs prior to visiting dental offices can help mitigate COVID-19 transmission, both to staff and patients. Ideally, the need for RATs should be incorporated into dental treatment screening procedures during the pandemic, and the frequency of the test should be increased. The latter applies to both dental staff and dental patients, as an individual who appears healthy may be an asymptomatic carrier [33]. Another preventive measure to reduce COVID-19 transmission is having patients gargle with mouthwash before having a dental procedure. The results of this study showed that most dentists always recommend to their patients that they gargle with mouthwash prior to dental treatment (Fig. 3A). In the present study, the most used mouthwash was povidone-iodine (PVP-I) (Fig. 3B). Research has yet to show a clinically effective reduction in the salivary load of SARS-CoV2 at a large population scale associated with gargling with mouthwashes, although gargling with chlorhexidine [34], 1–1.5% hydrogen peroxide [35], cetylpiridinium chloride (CPC) [36], or PVP-I [37] mouthwashes in advance of dental procedures has been reported to reduce viral loads in vitro, which in turn may inhibit COVID-19 transmission during dental procedures [38, 39]. At present, the recommended antimicrobial mouthwashes are chlorhexidine gluconate, cetylpyridinium chloride, PVP-I, and hydrogen peroxide [38]. Rubber dams can also be used to prevent viral transmission. Dentists are recommended to use a rubber dam whenever possible [17].

Since the pandemic, direct human − human interactions have been severely limited to prevent COVID-19 transmission. Due to available technologies, many employees, even health workers, can work remotely. Telemedicine involves a combination of current technologies, such as smartphones and the Internet, in addition to health workers’ expertise, to enable clinical examinations to be performed remotely as there is no direct contact with the patient, telemedicine reduces the potential risk of COVID-19 transmission [40]. Dental health workers can also provide oral health examinations (teledentistry) remotely.

Teledentistry is suitable for the management of minor dental complaints, as well as some more severe complaints. However, in some cases, it will be necessary for the patient to attend a dental clinic in person. As shown in Fig. 3C, a relatively high percentage of dentists in educational facilities (± 60%) perform teledentistry versus a comparatively low percentage in public (government) hospitals and private hospitals. Therefore, dentists in university hospitals are more aware of teledentistry than those in government and private hospitals [41, 42]. The results of this study highlight the value of teledentistry during the pandemic. The findings may also encourage greater take-up of teledentistry among dentists in all settings (i.e., public and private hospitals) in the future. In terms of entrance screening, this study is in line with the previous study in Japan. 96% of the respondents performed screening on symptoms and body temperature prior to dental treatment. However, in terms of infection control, only 43.1% of dentists stated that they would wear an N95 mask and only 35.5% of dentists would recommend patients rinse their mouth with mouthwash before dental treatment [43].

The limitation of this study is that the respondents only reached 18% (5,323) of the total number of dentists in Indonesia (29,510). However, the respondents have represented the locations of dentists in Indonesia. The results of this study can be used as a basis for policymakers to make the screening a standard dental treatment screening procedure during the covid pandemic. The purpose of this research has been achieved in accordance with the results obtained. From the results of the study, researchers can find out that dental practitioners in Indonesia have used updated pre-procedure dental treatment protocols during the COVID-19 pandemic.

## Conclusions

Almost all dentists in government hospitals, private hospitals, and university hospitals in Indonesia perform pre-procedure patient screening. There is an agreement between dental professionals in government hospitals, private hospitals, and dental schools about the need for patient screening procedures in dental practices prior to dental treatment during the COVID-19 pandemic. This paper may provide insight into dental practice during the COVID-19 pandemic for both dental organizations and the government, particularly the ministry of health.

## Data Availability

Further information on the data set and materials is available from the corresponding author upon reasonable request.

## Abbreviations

WHO: World Health Organization
SARS-CoV2: Severe acute respiratory syndrome coronavirus 2
EPA: United States Environmental Protection Agency
CDC: Center for Disease Control and Prevention
RAT: Rapid antigen test
ORs: Odds ratios
Cis: Confidence intervals
RT-qPCR: Reverse transcription-quantitative Polymerase Chain Reaction
PVP-I: Povidone-iodine
CPC: Cetylpiridinium chloride
